# Effect of Guanidinoacetic Acid on Production Performance, Serum Biochemistry, Meat Quality and Rumen Fermentation in Hu Sheep

**DOI:** 10.3390/ani14142052

**Published:** 2024-07-12

**Authors:** Huayun Jin, Zhijian Du, Xiaoyu Fan, Liwen Qin, Weiwei Liu, Yan Zhang, Jingnan Ren, Changchuan Ye, Qinghua Liu

**Affiliations:** Department of Animal Science, College of Animal Science, Fujian Agriculture and Forestry University, Fuzhou 350002, China; jhy001x@163.com (H.J.); 17607848230@163.com (Z.D.); 17852396033@163.com (X.F.); 18706011090@163.com (L.Q.); lww990609@163.com (W.L.); zhangyan2990@163.com (Y.Z.); 15392547223@163.com (J.R.)

**Keywords:** Hu sheep, guanidinoacetic acid, production performance, rumen fermentation, fatty acid metabolism

## Abstract

**Simple Summary:**

Recently, the escalating prices of feedstuff resources have posed significant challenges to animal husbandry, necessitating the creation of innovative approaches to mitigate its effects. GAA (also known as N-amidinoglycine) serves as the sole precursor for creatine synthesis. GAA could enhance growth performance and regulate fat deposition to improve meat quality. Current studies on GAA mainly center on pigs and chickens, with limited reports on its effects on sheep. In our study, we used weaned Hu sheep as subjects. We aimed to explore the impact of dietary GAA supplementation on production performance, serum biochemistry, meat quality and rumen fermentation. This study provides a theoretical foundation for utilizing GAA in sheep meat production. Our findings indicate that GAA enhances growth performance, rumen fermentation and fat accumulation. In addition, GAA might enhance the quality of sheep meat, which presents a promising prospect for meat sheep breeding.

**Abstract:**

Guanidinoacetic acid (GAA) can effectively improve the metabolism of energy and proteins by stimulating creatine biosynthesis. We present a study exploring the impact of GAA on production performance, serum biochemistry, meat quality and rumen fermentation in Hu sheep. A total of 144 weaned male Hu sheep (body weight 16.91 ± 3.1 kg) were randomly assigned to four groups with three replicates of twelve sheep in each group. The diets were supplemented with 0 (CON), 500 (GAA−1), 750 (GAA−2) and 1000 mg/kg (GAA−3) of GAA (weight of feed), respectively. After a comprehensive 90-day experimental period, we discovered that the supplementation of GAA had a remarkable impact on various muscle parameters. Specifically, it significantly enhanced the average daily growth (ADG) of the animals and improved the shear force and fiber diameter of the muscle, while also reducing the drip loss and muscle fiber density. Furthermore, the addition of GAA to the feed notably elevated the serum concentrations of high-density lipoprotein cholesterol (HDL−C), total protein (TP) and globulin (GLB), as well as the enzyme activity of superoxide dismutase (SOD) and glutathione peroxidase (GSH−Px). Concurrently, there was a decrease in the levels of triglycerides (TG) and malondialdehyde (MDA) in the serum. In addition, GAA decreased the pH and the acetate-to-propionate ratio and increased the total volatile fatty acids (TVFA) and ammoniacal nitrogen (NH_3_−N) levels of rumen fluid. Additionally, GAA upregulated acetyl-CoA carboxylase (ACC) gene expression in the Hu sheep’s muscles. In conclusion, our findings suggest that GAA supplementation not only enhances muscle quality but also positively affects serum biochemistry and ruminal metabolism, making it a potential candidate for improving the overall health and performance of Hu sheep.

## 1. Introduction

Recently, the escalating prices of feedstuff resources have significantly affected animal husbandry, particularly intensive sheep farming in China. This trend has posed significant challenges to the industry, necessitating the creation of innovative approaches to mitigate its effects. Guanidinoacetic acid (GAA) is a metabolic intermediate synthesized from arginine and glycine [1]. It has been reported that GAA could effectively enhance the metabolism of energy and proteins by stimulating creatine biosynthesis, thereby contributing to improved nutritional utilization and animal performance [2,3]. In monogastric animals, dietary supplement of GAA enhances feed intake and efficiency, growth performance, antioxidant status and meat quality [2,4,5]. Studies have found that adding GAA to the diet could have a regulatory effect on the growth performance, rumen fermentation and fat deposition of ruminants [6,7].

GAA is also known as N-amidinoglycine, which serves as the only precursor for creatine synthesis. As the primary stored form of phosphocreatine within skeletal muscle, creatine serves as a crucial component for recycling ATP and supporting various cellular functions. GAA and creatine are both amino acid derivatives derived from animals. Since plant proteins lack GAA and creatine, ruminants acquire creatine only through de novo synthesis [8]. In living organisms, endogenous creatine biosynthesis occurs via the kidney–liver axis. In this pathway, methionine, glycine and arginine are the substrates for the biosynthesis of creatine [9]. The high-energy phosphate bonds stored in muscles are crucial for muscle activity. Due to the considerable requirement of arginine for protein synthesis, endogenous creatine synthesis places a heavy metabolic burden on arginine and methionine [10]. Ardalan et al. found that post-ruminal GAA supplementation improved creatine availability in cattle [11]. Dietary supplementation with GAA in livestock (especially in broilers) can benefit animal growth performance, enhance muscle development and improve animal health [12,13,14]. A previous study suggested that GAA supplementation in broilers can enhance carcass yield and lower the levels of abdominal fat [15]. Li et al. found that dietary supplementation with GAA in finishing pigs raised pH45min and reduced drip loss and shear force [16]. Mendoza et al. revealed that GAA supplementation for gestating and lactating sows enhanced litter weight and weaning numbers without compromising their reproductive performance [17]. Recently, researchers have increasingly focused on the effects of GAA in ruminants. Studies conducted on bulls have revealed that GAA supplementation within the diet enhanced daily gain, nutrient digestion, blood creatine level and liver protein synthesis gene expression [18,19]. Another study found that dietary supplementation with GAA in lambs raised the concentrations of phosphocreatine and ATP in the muscle [20]. These findings indicated that GAA may act a pivotal role in promoting ruminant growth and overall health.

With the proposed conditions of use, GAA effectively improves the production performance of poultry and pigs without affecting consumer safety [21,22]. Currently, research on GAA in ruminants is scarce, and no safety concerns have been reported. In addition, few studies have been conducted on the use of GAA in sheep with a diet made primarily from plant-based proteins. Due to there being limited information on the effects of dietary GAA supplementation on sheep’s growth performance and fat deposition, there was a gap left in our understanding of its potential benefits and interactions within this nutritional context. Therefore, we conducted an experiment to explore the possibility of GAA application in lamb meat production. In this study, we aimed to explore the impacts of GAA on Hu sheep’s production, rumen fermentation and fatty acid metabolism. We believe that our result will provide insights into the potential benefits and optimal dosage of GAA in meat sheep production. Furthermore, this research will provide valuable insights into improving the overall welfare and productivity of livestock.

## 2. Materials and Methods

### 2.1. Moral Statement

The study was approved by the Fujian Agriculture and Forestry University Animal Care and Use Committee (Approval ID: PZCASFAFU23012).

### 2.2. Experimental Design and Feeding Management

Weaned Hu sheep were selected as experimental animals in this experiment. The experiments were conducted at the sheep farm of Longlin Husbandry Co., Ltd., Ningde City, China. This experiment was performed from March to June in 2023. A total of 144 weaned male Hu sheep (average initial body weight: 16.91 ± 3.10 kg; average age: 3 months) were used in a 90-day performance trial. The sheep were assigned to four treatments in a completely randomized block design. Within each block, the twelve sheep were randomly assigned to one of the four treatment groups using a random number generator to ensure unbiased allocation. Body weight was used as a block factor to control its potential impact on the experimental results. Similar body weight distribution was ensured within each block and sheep were randomly assigned to the groups. Each treatment was replicated three times across the different blocks, totaling 36 sheep per treatment (including control group) and 144 sheep in the entire study. The sheep were fed with a basal diet supplemented with 0 (CON group), 500 (GAA−1 group), 750 (GAA−2 group) and 1000 (GAA−3 group) mg·kg^−1^ GAA product (weight of feed), respectively. Table 1 showed the feed composition and nutrient levels for the experimental ration.

The sheep were fed at 08:00 and 16:00 daily. The GAA product labeled “Saint-Lo” (with GAA content over 50% with the remaining comprising a diluent) was acquired from Beijing Gendone Technology Co., Ltd. (Beijing, China). It was pre-mixed into the concentrate for feeding as detailed. Referring to a previous study [20] and our preliminary experiment, the sheep were given a seven-day adaptation period for the diet. The sheep were provided with feed and water ad libitum. Feed intake was tracked on a daily basis. Residual feed from the previous day of each treatment group was collected and weighed before feeding every morning. Disinfection and immunization were conducted according to routine procedures of the farm. As for foot-and-mouth disease, sheep receive their first immunization at 28–35 days of age, followed by a booster shot one month later and then booster immunizations every 4–6 months. As for brucellosis, immunization is conducted at 3–4 months of age. As for coenurosis, sheep receive their first immunization at 3–4 months of age, followed by a booster shot one month later and then annual booster immunizations.

### 2.3. Growth Performance

During each replication, sheep were weighed on the 90th day, and the total feed intake of each replicate was recorded. Average daily feed intake (ADFI), average daily gain (ADG) and feed/gain ratio (F/G) were calculated for each sheep.

### 2.4. Sample Collection

Three sheep from each group were randomly selected for fasting and weighing. Blood samples from the jugular vein were stored at 4 °C for 2 h, then centrifuged at 3000× *g* for 15 min to separate serum. The collected serum samples were stored at −20 °C for the determination of serum biochemical indexes and antioxidant indexes. Then, these sheep were slaughtered, sampled and analyzed. The longissimus dorsi was selected as the muscle that would be sampled for slicing and analysis. Except for the muscle tissue samples used for the slicing and the serum samples, all samples were refrigerated at −80 °C before detection. Measurement indexes included live weight, carcass weight, dressing percentage and eye muscle area. Dressing percentage is calculated as (carcass weight/live weight) × 100%. 

### 2.5. Meat Quality and Histological Analyses

Muscle pH was measured at three spots on the longissimus dorsi muscle using a hand-held pH meter (PHSJ−3F, Shanghai, China). Drip loss was determined by hanging a loin section from a 5 cm × 3 cm × 2 cm longissimus dorsi sample in a sealed plastic bag for 24 h at 4 °C [16]. Shear force was determined by cutting a 3 cm × 1 cm × 1 cm piece of dorsal longissimus muscle and testing it with a digital muscle tenderness tester (C-LM3B, Tenovo, Beijing, China). Intramuscular fat was measured by extraction with petroleum ether.

Muscle samples were fixed, embedded in paraffin, sectioned and mounted on slides. Then, the sections were deparaffinized, rehydrated and stained with hematoxylin and eosin. After glass coverslip mounting, the H&E-stained cross-sections were imaged using a microscope (Nikon Eclipse Ci-L, Tokyo, Japan). The histological analyses of muscle were in accordance with the method of Deng [24]. The muscle fiber diameter was measured by using Image-Pro Plus 6.0 analysis software after imaging, with millimeter as the standard unit, and the diameters of 5 muscle fibers from each slice were measured (mm). The total number of muscle fibers was measured by calculating the total number of muscle fibers in each image. The total muscle fiber area was measured by quantifying the total area (in mm^2^) of muscle fibers in each image. The formula for calculating muscle fiber density is Muscle Fiber Density (fibers/mm^2^) = Total Number of Muscle Fibers (fibers)/Total Area of Muscle Fibers (mm^2^).

### 2.6. Serum Biochemical Indexes and Antioxidant Capacity

The serum biochemical parameters were determined using a fully automatic biochemical analysis system (BS−200, Mindray Biomedical Electronics Co., Ltd., Shenzhen, China). The corresponding kits were purchased from Hunan Yonghe Sunshine Technology Co., Ltd., Changsha, China. A comprehensive set of biochemical parameters were measured, including total protein (TP), triglyceride (TG), albumin (ALB), globulin (GLB), high-density lipoprotein cholesterol (HDL−C), total cholesterol (TC) and low-density lipoprotein cholesterol (LDL−C).

Serum superoxide dismutase (SOD), serum glutathione peroxidase (GSH−PX) activity and serum malondialdehyde (MDA) content were measured by using the corresponding test kit (Shanghai Liquid Quality Testing Technology Co., Ltd., Shanghai, China).

### 2.7. Rumen Fermentation

The rumen fluid was collected immediately after slaughter (less than 20 min). We measured the pH of ruminal fluid samples immediately using a pH meter (PHS−25C; Hangzhou Qiwei Instrument Co., Ltd., Hangzhou, China). Then, the rumen fluid was collected in a 10 mL centrifuge tube after being filtered through four layers of sterile gauze. Then, the fluid was stored at −80 °C.

The contents of volatile fatty acids (VFA), including isobutyric acid, acetic acid, isovaleric acid, butyric acid and propionic acid, were measured using a gas chromatograph (Shimadzu GC 2010-FID, Tokyo, Japan) [25,26]. The gas chromatograph system was equipped with a column (KB-FFAP, Kromat, Delran, NJ, USA), and a flame ionization detector was operated at 250 °C. A 0.81 mL/min mobile phase using N_2_ was applied to the column. The column was operated at 80 °C.

Ammoniacal nitrogen (NH_3_−N) content was measured using phenol-sodium hypochlorite colorimetry, following the Broderick and Kang method [27].

### 2.8. Quantitative Real-Time PCR for Gene Expression

After slaughter, longissimus dorsi muscle samples were quickly removed from the Hu sheep. These samples were put into 2 mL sterilization centrifuge tubes and frozen in liquid nitrogen. These samples were stored at −80 °C for subsequent analysis.

Muscular total RNAs were isolated using the NucleoZol reagent (Gene, Düren, Germany) and cDNA was synthesized using reverse transcription reagent kits (Servicebio, G3330−100, Wuhan, China). Quantitative real-time PCR was performed with ABI 7300 Real-Time PCR System (Applied Biosystems, Foster City, CA, USA). Primer sequences for hormone-sensitive lipase (HSL), fatty acid synthase (FAS), acetyl-CoA carboxylase (ACC) and the reference gene β-Actin (ACTB) are listed in Table 2. Selected samples from each process were analyzed in triplicate, utilizing ACTB as the reference gene. RNA expression was quantified using the 2^−ΔΔCT^ method [28].

### 2.9. Statistics Analysis

Statistical analyses were undertaken using SPSS 27.0 (SPSS, Inc., Chicago, IL, USA). Data analysis was conducted using one-way analysis of variance (ANOVA). The CON group (without GAA treatment) was set as control. When the ANOVA indicated significant differences among groups, Tukey’s multiple range tests were further employed to determine which specific pairs of groups were significantly different. The results are presented as the mean ± standard deviation (SD) and significance was declared at *p* ≤ 0.05.

## 3. Results

### 3.1. Effects of GAA Supplementation on Growth Performance, Carcass Traits and Meat Quality

According to Table 3, dietary supplementation with 750 and 1000 mg·kg^−1^ GAA (GAA−2 and GAA−3 groups, respectively) significantly increased the ADG of Hu sheep compared to the CON group (*p* < 0.05). However, the F/G and ADFI among these groups did not show significantly differences (*p* > 0.05). 

The carcass weight of the GAA−3 group was higher compared to the CON group (*p* < 0.05). There were no significant differences among these groups in terms of slaughter weight or dressing percentage (Table 3).

**Figure 1:**
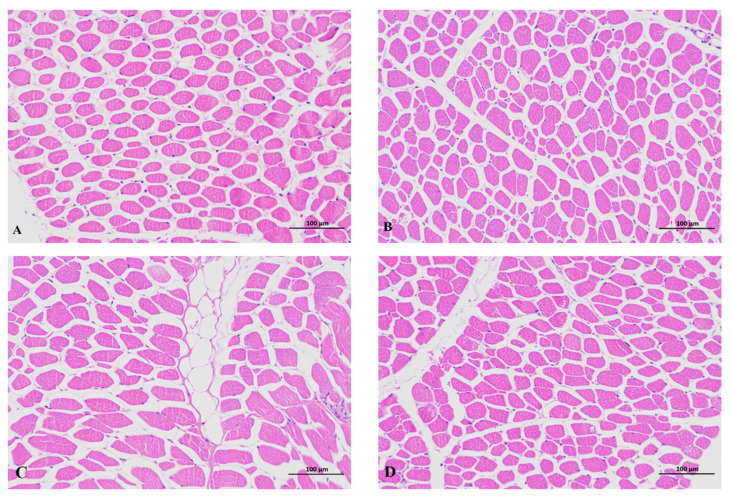
Microscopic images of the longissimus dorsi muscle showing cross-sections from the CON group (**A**), GAA−1 group (**B**), GAA−2 group (**C**), and GAA−3 group (**D**). The diets were supplemented with 0 (CON), 500 (GAA−1), 750 (GAA−2) and 1000 mg/kg (GAA−3) of GAA (weight of feed), respectively. The histological analyses of muscle were shown in Table 4.

According to Figure 1 and Table 4, dietary supplementation with 1000 mg·kg^−1^ GAA (GAA−3 group) reduced the muscle fiber density and the drip loss of meat, while the shear force and muscle fiber diameter increased (*p* < 0.05). No significant differences were observed in pH_24h_, crude fat ratio, total muscle fibers and total muscle fiber area among all groups (*p* > 0.05).

### 3.2. GAA Supplementation Alters Serum Lipid Profile and Protein Levels

**Figure 2:**
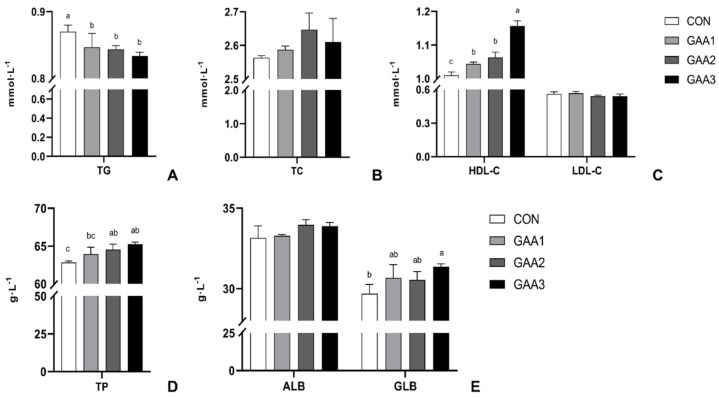
Serum biochemical parameters of Hu Sheep following GAA supplementation (**A**: the level of TG on serum; **B**: the level of TC on serum; **C**: the level of HDL−C and LDL−C on serum; **D**: the level of TP on serum; **E**: the level of ALB and GLB on serum). The error bar indicates standard deviation (data are from Table S1). Different letters indicate significant differences (*p* < 0.05). TG: Triglyceride; TC: total cholesterol; HDL−C: high-density lipoprotein cholesterol; LDL−C: low-density lipoprotein cholesterol; TP: total protein; ALB: albumin; GLB: globulin.

As shown in Figure 2, the dietary supplementation with GAA significantly reduced the level of TG (*p* < 0.05) in the sheep’s serum. The dietary supplementation with 1000 mg·kg^−1^ GAA (GAA−3 group) significantly increased the levels of HDL−C, TP and GLB (*p* < 0.05) in the serum. However, there were no significant differences in the contents of TC, LDL−C and ALB in the serum among these groups (*p* > 0.05).

### 3.3. GAA Supplementation Enhanced the Antioxidant Capacity on Serum

**Figure 3:**
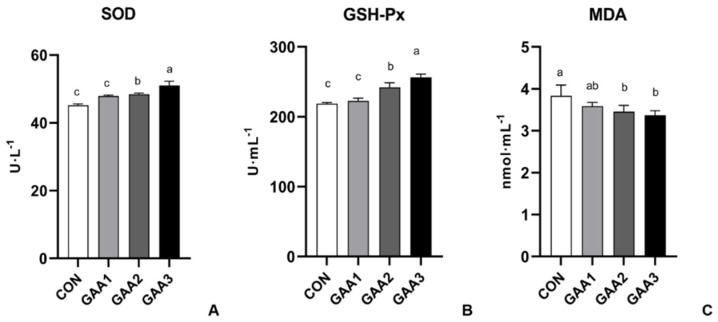
Serum antioxidant capacity of Hu sheep following GAA supplementation (**A**: the enzyme activity of SOD on serum; **B**: the enzyme activity of GSH−Px on serum; **C**: the level of MDA on serum). The error bar indicates standard deviation (data are from Table S2). Different letters indicate significant differences (*p* < 0.05). SOD: superoxide dismutase; GSH−PX: glutathione peroxidase; MDA: malondialdehyde.

According to Figure 3, the addition of GAA to feed would significantly enhance SOD activity in serum (*p* < 0.05). Furthermore, the dietary supplementation with 750 and 1000 mg·kg^−1^ GAA (GAA−2 and GAA−3 groups, respectively) significantly elevated the enzyme activity of GSH−Px (*p* < 0.05). Notably, the level of MDA significantly decreased compared to the CON group (*p* < 0.05), indicating a positive effect on antioxidant status.

### 3.4. Modulation of Rumen pH, VFA Profile, and NH_3_−N Content by GAA Supplementation

**Figure 4:**
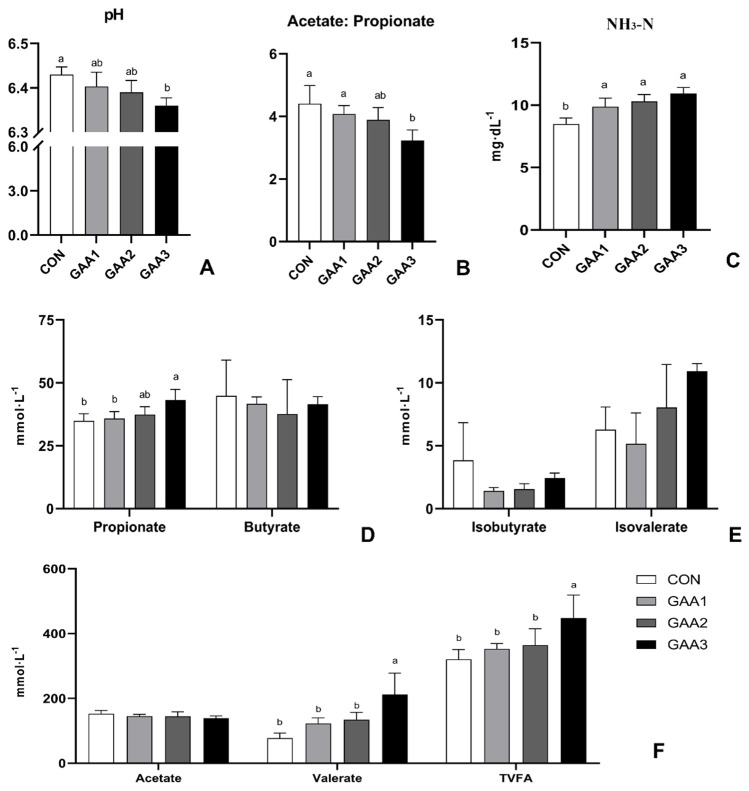
Rumen fermentation of Hu sheep following GAA supplementation (**A**: pH of rumen fluid; **B**: the ratio of acetate to propionate in rumen fluid; **C**: NH_3_−N content in rumen fluid; **D**–**F**: different VFA and TVFA contents in rumen fluid). The error bar indicates standard deviation (data are from Table S3). Different letters indicate significant differences (*p* < 0.05). NH_3_−N: Ammoniacal nitrogen; TVFA: Total volatile fatty acids.

As shown in Figure 4, ruminal pH exhibits a linear decrease with increasing GAA supplementation. The GAA−3 group showed a significantly lower pH compared to the CON group (*p* < 0.05). The total VFA concentration was significantly higher (*p* < 0.05) in the GAA−3 group compared to other groups. The valerate molar proportion increased (*p* < 0.05) in the GAA−3 group compared to the CON group. While the acetate molar proportion remained unaffected, the propionate molar proportion was greater (*p* < 0.05) in the GAA−3 group compared to the CON group. As a result, the acetate-to-propionate ratio decreased (*p* < 0.05) in the GAA−3 group compared to the CON group. As the supply of GAA increased, the molar proportions of valerate and the NH_3_−N content increased (*p* < 0.05), but the levels of butyrate, isobutyrate and isovalerate were unchanged.

### 3.5. GAA Supplementation Affects Fatty Acid Metabolism in Longissimus Dorsi Muscle

**Figure 5:**
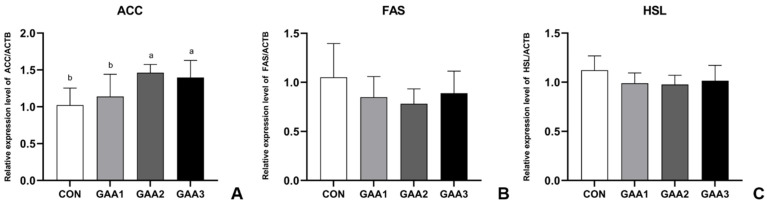
The expression of fatty acid metabolism-related genes (**A**: ACC, **B**: FAS, **C**: HSL) in the longissimus dorsi muscle of Hu sheep following GAA supplementation. ACTB (β-Actin) was set as reference gene. The error bar indicates standard deviation (data are from Table S4). Different letters indicate significant differences (*p* < 0.05). ACC: acetyl-CoA carboxylase; FAS: fatty acid synthase; HSL: sensitive lipase.

As illustrated in Figure 5A, the expression level of ACC mRNA in the longissimus dorsi muscle varied with increasing dietary GAA levels. Specifically, the GAA−2 group exhibited the highest expression, whereas the CON group had the lowest, with a significant difference (*p* < 0.05). According to Figure 5B,C, the expression levels of FAS and HSL mRNA in the longissimus dorsi muscle did not differ significantly (*p* > 0.05).

## 4. Discussion

Growth performance serves as a crucial metric for assessing the economic efficiency of livestock farming. Thus, enhancing animal growth performance holds great significance for driving the advancement of the farming industry. As a nutritional additive, GAA enhances energy metabolism, feed conversion efficiency and myoblast growth [10,29]. Jayaraman et al. discovered that dietary supplementation with GAA significantly increased the average daily weight gain and lean meat yield of fattening pigs [4]. Research indicates that GAA can effectively enhance the growth performance of livestock, especially by increasing daily weight gain and decreasing the feed-to-meat ratio [30]. Similarly, dietary supplementation with GAA has effectively improved growth performance and enhanced the growth of muscle and carcass weight in sheep [10]. Notably, dietary supplementation of GAA could promote skeletal muscle development [31]. Our study revealed that the addition of GAA to the sheep’s diet not only increased the average daily gain of Hu sheep, but also increased carcass weight. In addition, we discovered that providing sheep with a dietary GAA supplementation of 1000 mg·kg^−1^ increased the muscle fiber diameter of the longissimus dorsi muscle while decreasing muscle fiber density (Figure 1 and Table 4). These results indicated that a dietary GAA supplement might affect sheep muscle fiber traits. Since the number of primary and secondary myofibrils is fixed prenatally [32], the enhanced carcass weight of the GAA-fed sheep might result from modifications in myofibril size and muscle mass accumulation.

Muscle pH can reflect the changes in muscle glycolysis after slaughter, which affect meat drip loss, shear force and meat color [3,33]. It has been reported that the addition of 0.2% GAA had a significant effect on the reduction in the drip loss of the longest dorsal muscle of Jinjiang Bulls [34]. Kim et al. concluded that GAA supplementation delays pH decline, resulting in less protein denaturation and ultimately reduced drip loss [35]. Our results also indicated that the addition of 1000 mg·kg^−1^ GAA to the diet could significantly improve the drip loss of the longest dorsal muscle, which provides further data in support of the application of GAA in ruminants, particularly in sheep.

Skeletal muscle is primarily composed of muscle fibers, with a smaller percentage of connective tissue and adipose tissue. Thus, changes in muscle fibers affect indicators such as muscle shear force. In a previous study, it was found that muscle shear force and muscle fiber diameter showed a positive correlation [36]. Diet composition and nutritional levels could affect the diameter and density of muscle fibers [37]. Li et al. found that the GAA supplementation raised the final body weight, enhanced muscle mass and varied myofiber size distribution [10]. Changes in muscle fiber characteristics can to some extent explain changes in meat quality, for example, the size of myofibers can influence fiber bundle size and muscle growth prospects [38]. In our study, we found that the addition of GAA to the diet significantly increased the shear force of the longest dorsal muscle. The increase in shear force might be a potential adverse effect of GAA application, which might indicate tougher meat. We speculate that GAA enhances the protein utilization efficiency of the Hu sheep, which enlarges the muscle fiber diameter and affects the meat’s shear force. In addition, the muscle fiber characteristics of the Hu sheep were altered by the addition of GAA, with an increase in myofibril diameter and a decrease in myofibril density. These results suggested that the change in myofibril characteristics may be one of the reasons for the change in meat quality of the Hu sheep. The hardness of meat may also be related to intramuscular fat content, which did not significantly change in this study. Meanwhile, we need to consider both meat quality and the economic benefits of feeding. Thus, we still need to carry out further research to investigate the adverse effects of GAA on meat quality.

Serum biochemical indexes can intuitively reflect the metabolic level of animal bodies [39], which could also reflect the deposition of nutrients in the animal body. The levels of TC and TG in serum reflect the status of lipid metabolism in the body and are important indicators of adipose tissue development and deposition [40]. In this study, the serum concentrations of TC, TG, LDL−C and HDL−C were examined to ascertain the normality of lipid metabolism in the animals. HDL transports cholesterol from extrahepatic tissues to the liver for metabolism, while LDL carries cholesterol to peripheral tissues. Furthermore, serum LDL−C content is positively correlated with disease incidence [40]. In this study, supplementing with 1000 mg·kg^−1^ GAA significantly reduced the levels of TG in the serum of Hu sheep and increased the levels of HDL−C (Figure 2A,C). In addition, there were significant differences in the TC and LDL−C in serum among these groups (Figure 2B,C). This indicates that supplementing their diet with GAA can improve the serum lipid metabolism status of Hu sheep. Serum TP and ALB are closely related to protein metabolism in the body. They both perform crucial roles in maintaining vascular osmotic pressure, regulating pH levels and facilitating the transportation of nutritional metabolites [7]. Serum TP is an important indicator of liver function, and many enzymes in the body are proteins synthesized in the liver. A decrease in TP content indicates that the body’s metabolic capacity may be impaired [41]. Serum ALB is the main carrier for transporting nutrients. In this study, the levels of TP and GLB were notably elevated in the groups supplemented with GAA compared to the control group. However, ALB levels did not exhibit significant variations among the groups (Figure 2D,E). This result matches the outcomes of previous study conducted on Jinjiang Bulls [34]. Based on the enhancement of production performance and changes in muscle fibers, we believe that supplementing with GAA can improve the utilization of protein and other nutrients in Hu sheep.

The antioxidant enzymes SOD and GSH−Px play important roles in the in vivo antioxidant system, whereas MDA contents reflect the rate and intensity of lipid peroxidation in the body [42]. In our study, the addition of GAA led to elevated levels of SOD and GSH−Px and reduced MDA concentrations in the blood of Hu sheep (Figure 3), indicating that GAA enhanced the antioxidant capacity of sheep. It has been reported that the addition of GAA had a significant effect on the antioxidant indexes (SOD and GSH−Px) of Jinjiang Bulls [34]. Zhao et al. reported that supplementation GAA can decrease MDA contents of broilers [43]. GAA-related metabolites (creatine and arginine) have the potential to neutralize free radicals upon GAA consumption [44]. Creatine, which is one of the GAA products, has been shown to effectively eliminate O^2−^ [45]. Therefore, we suggest that GAA may stimulate sheep growth by enhancing their antioxidant potential.

The environment in the rumen can reflect bodily health, influencing digestion, absorption and nutrient utilization in meat-producing sheep. Our results indicate that GAA significantly impacts rumen pH, showing a decreasing trend and negatively correlated with total volatile fatty acids (TVFA) concentration. This is supported by the significant elevation in the molar ratio of propionic acid and valeric acid, when compared to the control group (Figure 4D,F). The changes in rumen pH are associated with increased or decreased concentrations of VFA [46]. VFA accumulation can lead to a decrease in rumen pH, with acetate and propionate being the main influences [47]. Acetic acid can be converted into Acetyl-CoA and is further involved in energy metabolism, fatty acid synthesis and other processes. Propionic acid is linked to carbohydrate metabolism and would inhibit cholesterol synthesis. Butyric acid is converted into β-Hydroxybutyric acid participates in the metabolic activities of tissues such as the liver and muscles to provide the energy needed by the body [48,49]. Our study found that the concentration of propionic acid in the rumen fluid showed an upward trend, which is consistent with the research reports of Li [18]. A significant increase in the rumen propionic acid molar ratio resulted in a corresponding decrease in the acetate-to-propionic acid ratio, indicating that GAA induced a shift in the rumen fermentation pattern towards increased propionic acid production. The increase in TVFA suggests that GAA may promote the synthesis of microbial proteins within the ruminal environment of Hu sheep. VFA is the main energy source for ruminants, providing more than 70% of the total energy needs of ruminants, and is also the main source of carbohydrates for rumen microorganisms [50]. We believe that supplemental feeding of GAA can elevate the VFA content in the rumen of Hu sheep, thereby enhancing energy availability to the body and ultimately resulting in improved weight gain and carcass quality. NH_3_−N is an apparent indicator of rumen nitrogen degradation in ruminants. It is both the end product of nitrogenous substance degradation in the diet and the raw material for the microbial synthesis of bacterial proteins [51]. In the present study, the levels of NH_3_−N in the experimental groups were significantly higher than that in the CON group, and the GAA−3 group exhibited the highest NH_3_−N content (Figure 4C). We hypothesized that the dietary supplementation of GAA in Hu sheep would enhance protein utilization and increase the accumulation of rumen decomposition products. This might lead to an increase in levels of NH_3_−N. Based on the above results, we believe that GAA supplementation can improve rumen fermentation by increasing TVFA, propionate and other metabolites, as well as enhancing specific serum biochemical indicators (including enzyme activity of SOD and GSH−Px and the level of MDA) in Hu sheep. This suggests an improved energy utilization efficiency, which likely contributes to changes in muscle fiber characteristics and ultimately improved production performance.

Intramuscular fat (IMF) is deposited in the longest muscle of the back, infraspinatus, biceps femoris and other muscle parts, and it directly affects the level of meat quality. IMF improves tenderness, color, juiciness, texture and flavor. These indicators are crucial for assessing meat quality [52]. Research has shown that GAA may reduce fat deposition by affecting enzyme activities and regulating lipid metabolism [53]. When considering the influence of diet on IMF content, ruminants exhibit a lesser degree of sensitivity compared to monogastric animals. In our study, we selected the longissimus dorsi muscle to investigate the effect of related gene expression on IMF deposition. Acetyl-CoA serves as a crucial intermediate metabolite in the intricate process of fatty acid metabolism. Acetyl-CoA carboxylase (ACC) serves as the initial rate-limiting enzyme in de novo fatty acid synthesis. ACC localizes in the cytosol and catalyzes the conversion of acetyl-CoA to malonyl-CoA, which is subsequently utilized by fatty acid synthase (FAS) to generate long-chain saturated fatty acids [54]. FAS is closely related to the content of fatty acids in muscle and fat. The increase in FAS expression is often accompanied by the increase in triglyceride content in the body, leading to the accumulation of adipose tissue [55,56]. The hormone-sensitive lipase (HSL) gene is an enzyme related to fatty acid hydrolysis. It can hydrolyze triglycerides into non-esterified free fatty acids and glycerol, which plays a central role in regulating fat deposition [57]. We found that the expression level of ACC in the longissimus dorsi muscle of Hu sheep was upregulated in the experimental group (Figure 5A). However, we found no significant differences in FAS and HSL among the experimental groups and the CON group (Figure 5B,C). These findings suggested that GAA stimulated the synthesis of fatty acids in the muscle tissue of Hu sheep via the enhanced expression of ACC in the muscle, thereby promoting the development of intramuscular fat. Based on the observed changes in serum TG and HDL−C levels, we hypothesize that supplementing with GAA may have increased the expression of ACC in the longissimus dorsi muscle. This potential increase in ACC expression could be a contributing factor to the decreased level of triglycerides in serum, leading to greater storage of triglycerides in adipose tissue.

## 5. Conclusions

This study demonstrates the potential of dietary GAA supplementation to enhance the production performance, serum biochemistry, meat quality and rumen fermentation of Hu sheep. By enhancing the energy supply efficiency and antioxidant potential of Hu sheep, the supplementation of GAA significantly improved their production performance and muscle fiber characteristics. This was possibly associated with the increase in VFA as it plays a major part of providing energy in ruminants. A potential adverse effect of GAA application was found in this study in the form of an increase in shear force, which might indicate tougher meat. Meanwhile, GAA was able to increase the expression of ACC and might enhance the quality of sheep meat. Our results indicate that supplementing the diet with 1000 mg·kg^−1^ of GAA was the most effective in improving the production performance of fattened Hu sheep.

## Figures and Tables

**Table 1:** Experimental diet composition and nutrient level (Dry matter basis, %). The nutritional standards refer to the nutritional requirements for sheep according to NRC (2007) [23].

Dietary Ingredient	Contents
Bamboo shoot shells silage	60
Corn	17
Bran	12
Soybean meal	6
NaCl	1
CaHCO_3_	1.4
NaHCO_3_	1.2
Premix ^(1)^	1.4
Total:	100
Nutrient levels ^(2)^	
Metabolizable energy (MJ/Kg)	9.68
Crude protein	11.55
Neutral detergent fiber	58.46
Acid detergent fiber	36.32
Calcium	0.75
Phosphorus	0.50

^(1)^ The premix supplied Cu 20.0 mg, Fe 75.0 mg, Mn 30.0 mg, Zn 80.0 mg, I 1 mg, Se 0.50 mg, Vitamin A 20,000 IU, Vitamin D 5000 IU, and Vitamin E 50.0 mg per kg of diet. ^(2)^ Metabolizable energy (ME) were calculated values. The values of CP (crude protein), NDF (neutral detergent fiber), ADF (acid detergent fiber), Ca (calcium) and P (phosphorus) were measured and recorded. CP was determined by the Kjeldahl method; NDF and ADF were measured using the filter bag technique; Ca was determined by the ethylenediaminetetraacetic acid (EDTA) complexometric titration method; and P was measured by spectrophotometry at a wavelength of 400 nm with the molybdenum blue reaction.

**Table 2:** Nucleotide sequences of primers for quantitative real-time PCR assay.

Items	Primer Sequences (5′−3′)	Genebank Number	Product Length (bp)
ACC	F: CCTGTCCGCCATTGACAT	NM_001009256.1	171
	R: TAGGCGATATAAGCCCTTCG		
FAS	F: CAGTCGGTTGGATCGAGCAT	XM_015098375.1	151
	R: AGAAGGAGGGTGGCTTTTGG		
HSL	F: TACAAACGCAACGAGACTGG	NM_001128154.1	172
	R: ACGATAGCACCTGGATCTCG		
ACTB	F: CCCTGGAGAAGAGCTACGAG	NM_001009784.3	131
	R: GGTAGTTTCGTGAATGCCGC		

**Table 3:** Growth performance and carcass traits of Hu sheep following GAA supplementation.

Items	Groups				*p*-Value
	CON	GAA−1	GAA−2	GAA−3	
Growth performance					
Initial BW (kg)	17.45 ± 2.53	17.66 ± 3.28	17.99 ± 3.04	17.71 ± 2.79	0.960
Final BW (kg)	28.05 ± 2.60	28.75 ± 3.01	29.45 ± 3.65	29.42 ± 3.20	0.872
ADFI (kg)	1.17 ± 0.17	1.17 ± 0.22	1.26 ± 0.21	1.24 ± 0.23	0.934
ADG (g)	117.85 ± 1.57 ^b^	123.31 ± 3.76 ^ab^	127.43 ± 3.24 ^a^	130.12 ± 2.39 ^a^	0.021
F/G	9.71 ± 1.41	9.51 ± 1.81	10.15 ± 1.71	9.95 ± 1.85	0.969
Carcass traits					
Live weight (kg)	28.50 ± 0.50	28.50 ± 0.50	28.83 ± 0.29	29.00 ± 0.50	0.482
Carcass weight (kg)	12.35 ± 0.10 ^b^	12.35 ± 0.30 ^b^	12.45 ± 0.26 ^b^	13.17 ± 0.40 ^a^	0.023
Dressing percentage (%)	43.34 ± 1.11	43.34 ± 1.26	43.19 ± 1.25	45.40 ± 1.14	0.147
Eye muscle area (cm^2^)	11.67 ± 1.38	11.47 ± 1.06	13.25 ± 0.90	12.92 ± 1.76	0.318

BW, body weight; ADFI, average daily feed intake; ADG, average daily gain; F/G, feed/gain ratio. The results are presented as the mean ± SD. Different letters indicate significant differences (*p* < 0.05).

**Table 4:** Meat quality characteristics of Hu sheep following GAA supplementation.

Items	Groups				*p*-Value
	CON	GAA−1	GAA−2	GAA−3	
pH_24h_	5.22 ± 0.21	5.27 ± 0.19	5.36 ± 0.17	5.38 ± 0.40	0.890
Drip loss (%)	7.15 ± 2.85 ^a^	6.48 ± 1.52 ^ab^	5.01 ± 0.93 ^ab^	3.41 ± 0.55 ^b^	0.027
Shear force (N)	17.21 ± 0.9 ^b^	21.97 ± 1.55 ^ab^	23.49 ± 2.21 ^a^	25.71 ± 1.66 ^a^	0.034
Intramuscular fat (%)	5.42 ± 0.66	6.00 ± 0.75	6.33 ± 0.75	6.58 ± 0.82	0.328
Muscle fiber diameter (um)	27.77 ± 3.81 ^b^	28.08 ± 2.73 ^ab^	30.46 ± 3.32 ^a^	30.42 ± 3.50 ^a^	0.048
Total muscle fibers (N)	225.44 ± 46.75	202.56 ± 81.15	171.11 ± 25.77	213 ± 25.75	0.145
Total muscle fiber area (mm^2^)	0.15 ± 0.01	0.14 ± 0.04	0.16 ± 0.03	0.15 ± 0.01	0.362
Muscle fiber density (N/mm^2^)	1550.10 ± 298.57 ^a^	1375.38 ± 298.47 ^a^	1468.34 ± 161.05 ^a^	1089.78 ± 2 69.89 ^b^	0.005

The results are presented as the mean ± SD. Different letters indicate significant differences (*p* < 0.05).

## Data Availability

Most of the data generated or analyzed in this study are presented in this published article or its supplementary information. Additional data not included here are accessible upon reasonable request to the corresponding author.

## Supplementary Materials

The following supporting information can be downloaded at: https://www.mdpi.com/article/10.3390/ani14142052/s1. Table S1: Effect of GAA on serum biochemistry of Hu sheep; Table S2: Effect of GAA on serum antioxidant capacity of Hu sheep; Table S3: Effect of GAA on rumen fermentation of Hu sheep; Table S4: Effect of GAA on fatty acid metabolism-related genes in the longissimus dorsi.
