# Supplementary material for: Effect of Guanidinoacetic Acid on Production Performance, Serum Biochemistry, Meat Quality and Rumen Fermentation in Hu Sheep
Source: Animals (Basel). 2024 Jul 12;14(14):2052. doi: 10.3390/ani14142052 (PMC11273408; doi:10.3390/ani14142052)
Supplement: Supplementary file 1 [file animals-14-02052-s001.zip › animals-3015061-supplementary.pdf]

Table S1. Effect of GAA on serum biochemistry of Hu sheep.

Items	Groups				*P-value*
	CON	GAA-1	GAA-2	GAA-3	
TP ($g\cdot L^{-1}$)	62.85 ± 0.20^{c}	63.96 ± 0.90^{bc}	64.52 ± 0.74^{ab}	65.25 ± 0.30^{ab}	0.008
ALB ($g\cdot L^{-1}$)	33.15 ± 0.76	33.29 ± 0.08	33.97 ± 0.33	33.89 ± 0.23	0.110
GLB ($g\cdot L^{-1}$)	29.7 ± 0.57^{b}	30.67 ± 0.83^{ab}	30.56 ± 0.51^{ab}	31.36 ± 0.17^{a}	0.043
ALB/GLB	1.11 ± 0.05	1.09 ± 0.03	1.11 ± 0.02	1.08 ± 0.01	0.386
TG ($mmol\cdot L^{-1}$)	0.87 ± 0.01^{a}	0.85 ± 0.02^{b}	0.84 ± 0.01^{b}	0.83 ± 0.01^{b}	0.034
TC ($mmol\cdot L^{-1}$)	2.56 ± 0.01	2.59 ± 0.01	2.65 ± 0.05	2.61 ± 0.07	0.190
HDL-C ($mmol\cdot L^{-1}$)	1.01 ± 0.01^{c}	1.04 ± 0.01^{b}	1.06 ± 0.02^{b}	1.16 ± 0.02^{a}	<0.010
LDL-C ($mmol\cdot L^{-1}$)	0.56 ± 0.02	0.57 ± 0.02	0.54 ± 0.01	0.54 ± 0.02	0.193

The results are presented as the mean ± SD. Different letters indicate significant differences ($P < 0.05$).

Table S2. Effect of GAA on serum antioxidant capacity of Hu sheep.

Items	Groups				*P-value*
	CON	GAA-1	GAA-2	GAA-3	
MDA ($nmol\cdot mL^{-1}$)	3.84 ± 0.25^{a}	3.59 ± 0.09^{ab}	3.46 ± 0.15^{b}	3.37 ± 0.11^{b}	0.036
GSH-Px ($U\cdot mL^{-1}$)	218.9 ± 1.45^{c}	222.91 ± 3.63^{c}	242.03 ± 6.66^{b}	256.46 ± 4.59^{a}	<0.010
SOD ($U\cdot L^{-1}$)	45.17 ± 0.41^{c}	47.92 ± 0.23^{b}	48.44 ± 0.33^{b}	51.04 ± 1.21^{a}	<0.010

The results are presented as the mean ± SD. Different letters indicate significant differences ($P < 0.05$).

Table S3. Effect of GAA on rumen fermentation of Hu sheep.

Items	Groups				*P-value*
	CON	GAA-1	GAA-2	GAA-3	
pH	6.43 ± 0.02^{a}	6.40 ± 0.03^{ab}	6.39 ± 0.03^{ab}	6.36 ± 0.02^{b}	*0.043*
Acetate ($mmol\cdot L^{-1}$)	153.04 ± 9.82	145.56 ± 5.38	145.13 ± 13.5	138.59 ± 7.51	*0.386*
Propionate ($mmol\cdot L^{-1}$)	34.92 ± 2.78^{b}	35.82 ± 2.8^{b}	37.4 ± 3.13^{ab}	43.16 ± 4.25^{a}	*0.026*
Isobutyrate ($mmol\cdot L^{-1}$)	3.84 ± 2.99	1.42 ± 0.26	1.55 ± 0.43	2.43 ± 0.40	*0.267*
Butyrate ($mmol\cdot L^{-1}$)	44.83 ± 14.21	41.63 ± 2.76	37.63 ± 13.64	41.55 ± 2.99	*0.854*
Isovalerate ($mmol\cdot L^{-1}$)	6.28 ± 1.80	5.16 ± 2.44	8.04 ± 3.41	10.91 ± 0.62	*0.067*
Valerate ($mmol\cdot L^{-1}$)	77.73 ± 15.5^{b}	123.07 ± 16.97^{b}	134.45 ± 22.74^{b}	211.51 ± 66.1^{a}	*0.013*
Total VFA ($mmol\cdot L^{-1}$)	320.64 ± 17.3^{b}	352.66 ± 9.59^{b}	364.21 ± 29.39^{b}	448.14 ± 40.99^{a}	*0.037*
Acetate: Propionate	4.41 ± 0.58^{a}	4.08 ± 0.27^{a}	3.89 ± 0.39^{ab}	3.23 ± 0.33^{b}	*0.043*
NH_3-N ($mg\cdot dL^{-1}$)	8.49 ± 0.48^{b}	9.88 ± 0.67^{a}	10.31 ± 0.55^{a}	10.92 ± 0.50^{a}	*0.004*

The results are presented as the mean ± SD. Different letters indicate significant differences ($P < 0.05$).

Table S4. Effect of GAA on fatty acid metabolism-related genes in the longissimus dorsi muscle of Hu sheep.

**Items**		**Groups**				*P-value*
		**CON**	**GAA-1**	**GAA-2**	GAA-3	
Fatty acid production	ACC	1.02 ± 0.23b	1.14 ± 0.3b	1.46 ± 0.11a	1.40 ± 0.23a	*0.001*
	FAS	1.05 ± 0.34	0.85 ± 0.21	0.78 ± 0.15	0.89 ± 0.23	*0.134*
Fatty acid hydrolysis	HSL	1.12 ± 0.15	0.99 ± 0.11	0.98 ± 0.09	1.02 ± 0.16	*0.088*

The results are presented as the mean ± SD. Different letters indicate significant differences ($P < 0.05$).
